# Effect of Bromocriptine-QR (a Quick-Release Formulation of Bromocriptine Mesylate) on Major Adverse Cardiovascular Events in Type 2 Diabetes Subjects

**DOI:** 10.1161/JAHA.112.002279

**Published:** 2012-10-25

**Authors:** J. Michael Gaziano, Anthony H. Cincotta, Aaron Vinik, Lawrence Blonde, Nancy Bohannon, Richard Scranton

**Affiliations:** Massachusetts Veterans Epidemiology, Research and Information Center, Veterans Affairs Healthcare System Boston, Divisions of Aging, Cardiology, Preventive Medicine, Department of Medicine, Brigham and Women's Hospital, Harvard Medical School, Boston, MA (J.M.G.); VeroScience LLC, Tiverton, RI (A.H.C., R.S.); The Strelitz Diabetes Center and Neuroendocrine Unit, Norfolk, VA (A.V.); Department of Endocrinology, Ochsner Medical Center, New Orleans, LA (L.B.); St. Luke's Hospital, San Francisco, CA (N.B.)

**Keywords:** bromocriptine, circadian rhythm, Cycloset, diabetes mellitus type 2, infarction

## Abstract

**Background:**

Bromocriptine-QR (a quick-release formulation of bromocriptine mesylate), a dopamine D2 receptor agonist, is a US Food and Drug Administrration–approved treatment for type 2 diabetes mellitus (T2DM). A 3070-subject randomized trial demonstrated a significant, 40% reduction in relative risk among bromocriptine-QR-treated subjects in a prespecified composite cardiovascular (CV) end point that included ischemic-related (myocardial infarction and stroke) and nonischemic-related (hospitalization for unstable angina, congestive heart failure [CHF], or revascularization surgery) end points, but did not include cardiovascular death as a component of this composite. The present investigation was undertaken to more critically evaluate the impact of bromocriptine-QR on cardiovascular outcomes in this study subject population by (1) including CV death in the above-described original composite analysis and then stratifying this new analysis on the basis of multiple demographic subgroups and (2) analyzing the influence of this intervention on only the “hard” CV end points of myocardial infarction, stroke, and CV death (major adverse cardiovascular events [MACEs]).

**Methods and Results:**

Three thousand seventy T2DM subjects on stable doses of ≤2 antidiabetes medications (including insulin) with HbA1c ≤10.0 (average baseline HbA1c=7.0) were randomized 2:1 to bromocriptine-QR (1.6 to 4.8 mg/day) or placebo for a 52-week treatment period. Subjects with heart failure (New York Heart Classes I and II) and precedent myocardial infarction or revascularization surgery were allowed to participate in the trial. Study outcomes included time to first event for each of the 2 CV composite end points described above. The relative risk comparing bromocriptine-QR with the control for the cardiovascular outcomes was estimated as a hazard ratio with 95% confidence interval on the basis of Cox proportional hazards regression. The statistical significance of any between-group difference in the cumulative percentage of CV events over time (derived from a Kaplan–Meier curve) was determined by a log-rank test on the intention-to-treat population. Study subjects were in reasonable metabolic control, with an average baseline HbA1c of 7.0±1.1, blood pressure of 128/76±14/9, and total and LDL cholesterol of 179±42 and 98±32, respectively, with 88%, 77%, and 69% of subjects being treated with antidiabetic, antihypertensive, and antihyperlipidemic agents, respectively. Ninety-one percent of the expected person-year outcome ascertainment was obtained in this study. Respecting the CV-inclusive composite cardiovascular end point, there were 39 events (1.9%) among 2054 bromocriptine-QR-treated subjects versus 33 events (3.2%) among 1016 placebo subjects, yielding a significant, 39% reduction in relative risk in this end point with bromocriptine-QR exposure (*P*=0.0346; log-rank test) that was not influenced by age, sex, race, body mass index, duration of diabetes, or preexisting cardiovascular disease. In addition, regarding the MACE end point, there were 14 events (0.7%) among 2054 bromocriptine-QR-treated subjects and 15 events (1.5%) among 1016 placebo-treated subjects, yielding a significant, 52% reduction in relative risk in this end point with bromocriptine-QR exposure (*P*<0.05; log-rank test).

**Conclusions:**

These findings reaffirm and extend the original observation of relative risk reduction in cardiovascular adverse events among type 2 diabetes subjects treated with bromocriptine-QR and suggest that further investigation into this impact of bromocriptine-QR is warranted.

**Clinical Trial Registration:**

URL: http://clinicaltrials.gov. Unique Identifier: NCT00377676

## Introduction

Bromocriptine-QR (a quick-release formulation of bromocriptine mesylate), a dopamine D2 receptor agonist that reduces postprandial hyperglycemia without raising plasma insulin levels, was approved by the US Food and Drug Administration for the treatment of hyperglycemia in type 2 diabetes mellitus (T2DM) patients in May 2009 under the trade name Cycloset.^1–3^ The primary end points of the 1-year randomized, controlled Cycloset Safety Trial were the overall and cardiovascular (CV) safety of bromocriptine-QR versus placebo in a large population of subjects with T2DM.^4^ The composite cardiovascular event end point included the ischemic-related end points of myocardial infarction (MI) and stroke as well as the nonischemic end points of hospitalization for unstable angina, revascularization surgery, or congestive heart failure (composite cardiovascular end point). Analyses to assess various baseline covariates and the composite cardiovascular end point were prespecified in the study's statistical analysis plan.^4^ A prespecified primary objective of the study was to assess the cardiovascular benefit of bromocriptine-QR exposure subsequent to the demonstration of noninferiority to placebo.^4^ A sponsor-independent, blinded Event Adjudication Committee (EAC) adjudicated all serious adverse events and the inclusion of cardiovascular serious adverse events into the composite cardiovascular end point. The trial results demonstrated a statistically significant, 40% reduction in relative risk for serious cardiovascular events in subjects exposed to bromocriptine-QR versus placebo.^5^

The Cycloset Safety Trial, however, did not include CV death in this prespecified composite cardiovascular end point, thus limiting the interpretation of results from that prespecified analysis, in part because the impact of bromocriptine-QR on the “hard” ischemic CV composite end point of MI, stroke, and CV death remains unknown. To provide additional insight into the cardiovascular impact of bromocriptine-QR in T2DM subjects, we conducted 2 additional post hoc cardiovascular end-point analyses that each included CV death. The first included CV death as an additional component to the prespecified composite cardiovascular end point described above (CV death-inclusive composite cardiovascular end point) and assessed the influence of bromocriptine-QR on the CV death-inclusive composite cardiovascular end point stratified further by various baseline demographic subgroups. The second analysis evaluated only the “hard” ischemic end points of MI, stroke, and CV death (major adverse cardiovascular event [MACE] analysis), a standard and critically important end point for CV outcomes trials.

## Methods

### Study Design

The protocol for the Cycloset Safety Trial has been previously published.^4,5^ Briefly, this was a 12-month multicenter, placebo-controlled, double-blind, parallel-group safety and efficacy study in outpatient subjects with type 2 diabetes mellitus. Following a 2-week lead-in period, subjects were randomized in a 2:1 ratio to the usual diabetes treatment (see below) plus Cycloset or the usual diabetes treatment plus placebo. Subjects were recruited from general practice and diabetes clinics across 74 clinical centers in the United States and Puerto Rico, including 19 Veteran Affairs Medical Centers.

Eligible patients had type 2 diabetes as defined by the 2004 American Diabetes Association (ADA) guidelines,^6^ were between 30 and 80 years old, had a body mass index <43 kg/m^2^, and an HbA1c ≤10.0%. Subjects with New York Heart Classifications I and II heart failure were allowed to participate in the study, as were subjects with a history of myocardial infarction or coronary revascularization occurring >6 months before enrollment.

Subjects were required to be on a stable antihyperglycemic regimen for ≥30 days prior to randomization, consisting of either lifestyle interventions of medical nutrition therapy and appropriately prescribed physical activity, oral antihyperglycemic agents (≤2) or insulin (alone or with ≤1 oral antihyperglycemic agent), the usual diabetes treatment. During the first 6 weeks of the trial, the study drug was titrated weekly by adding 1 tablet per week (0.8 mg bromocriptine-QR per tablet) until a maximum tolerated daily dose between 2 and 6 tablets (1.6 to 4.8 mg/day of bromocriptine-QR) was achieved. The study drug was taken once daily with the morning meal, within 2 hours of waking. Patients were required to continue their usual other antihyperglycemic treatments during the first 3 months of the study. However, the dosages of the oral agents or insulin could be modified as deemed appropriate by the study site investigator to achieve the target glycemic goals in the 2004 ADA treatment recommendations.^7^ After 3 months, alterations in the diabetes treatment regimen were allowed if deemed necessary by the study site investigator; however, these changes could not include additions that resulted in a final regimen that exceeded 2 oral agents or insulin plus 1 oral agent, exclusive of the study drug.

During the titration phase, subjects received a weekly phone call and had office visits during weeks 3 and 6. After titration, subjects were seen during week 12 and then every 3 months until the study's end (week 52) or early termination. Subjects were contacted 30 days after completion of the study drug to record any subsequent adverse events. Physical examinations and laboratory assessments of hematology and blood chemistry, including HbA1c, lipids, and liver function tests, as well as urine analyses were obtained at baseline, week 24, and week 52. The study protocol was approved by site-specific or central institutional review boards.

### Outcomes Measures and Statistical Analyses

The CV death-inclusive composite cardiovascular end point of the present investigation was defined as the study-prespecified composite cardiovascular end point (the first event of the composite of myocardial infarction, stroke, coronary revascularization, or hospitalization for angina or CHF) plus the event of CV death (prespecified by the EAC of the Cycloset Safety Trial as death due to sudden cardiac death, MI, unstable angina, or other coronary artery disease [CAD]; vascular death [eg, stroke, arterial embolism, pulmonary embolism, ruptured aortic aneurysm, or dissection]; CHF; or cardiac arrhythmia). All deaths were assessed by the EAC, consisting of 2 cardiologists and an endocrinologist who reviewed medical records to determine whether death was cardiac related. The committee also determined whether serious adverse events (SAEs) should be included in the category of the prespecified cardiovascular end point.

All statistical analyses were conducted on the intention-to-treat (ITT) population. Total observed person-year outcome ascertainment for the study end points was derived from the following subject dispositions: (1) subjects who completed the 52-week (plus 30-day follow-up) trial on the study drug, (2) subjects who withdrew from study treatment before week 52 but remained in the study (ie, on study, off drug, and completing all study visits per protocol) through week 52 plus the 30-day follow-up period, (3) subjects who withdrew from the study before week 52 but provided end-point data on a prespecified case report form at their projected week 52 termination visit point, and (4) subjects who early-terminated the study due to a censoring (CV end point) event or other reason. Statistical methods for the analyses of the CV death-inclusive composite cardiovascular end point are essentially the same as previously reported for the prespecified composite cardiovascular end point.^4,5^ Analysis of the composite cardiovascular end point was conducted, adjusting for baseline covariates including history of stroke and cardiovascular revascularization surgery and study site. Time to first CV death-inclusive composite cardiovascular event was further analyzed within the following subgroups: sex (male versus female), age (≤65 versus >65 years), race (white versus nonwhite), preexisting macrovascular disease (including medical history of myocardial infarction, stroke, CAD, angina, coronary revascularization, peripheral vascular disease/revascularization), mean body mass index (BMI; ≤32 versus >32 kg/m^2^), and duration of diabetes (≤8 versus >8 years), and interaction *P* values for these subgroups were obtained as well. A Cox regression model was used to determine the effect of the various demographic subgroups and their treatment interaction. The main effect of each subgroup parameter was estimated from the model along with the treatment parameter estimate, and the treatment by subgroup interaction parameter was estimated with the associated *P* value presented.

Likewise, for the MACE analysis (occurrence of stroke, myocardial infarction, or CV death) the relative risk comparing bromocriptine-QR with the control group was estimated as a hazard ratio (HR) with a 95% confidence interval (CI) on the basis of Cox proportional hazards regression. Two-sided *P* values were calculated with the use of log-rank tests, unadjusted for multiple testing to assess statistical significance of between-group differences in the cumulative percentage in cardiovascular events generated from the Kaplan–Meier curves for the CV-death-inclusive composite cardiovascular end point and the MACE end point. All analyses were conducted using SAS statistical software version 9.2 (Cary, NC). The authors had full access to and take full responsibility for the integrity of the data.

## Results

**Figure 1.**
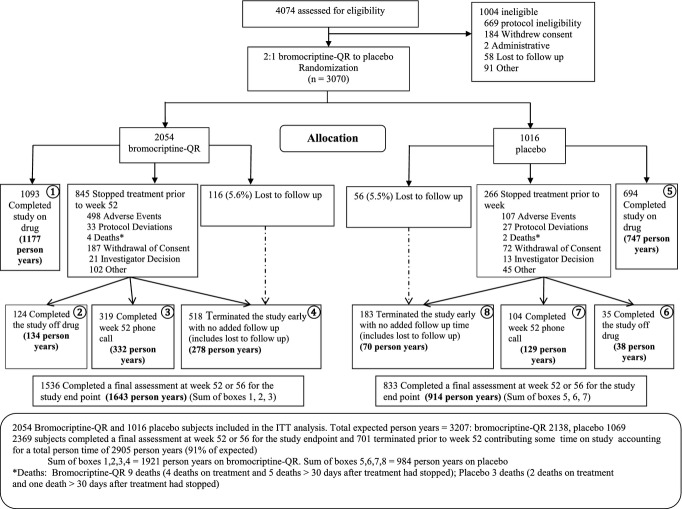
Subject disposition. ITT indicates intention-to-treat.

A total of 4074 subjects were screened, and 1004 subjects were excluded for not meeting eligibility criteria, withdrawal of consent, or other reasons, yielding 3070 subjects randomized to study treatment.^5^ In total, 91% of the planned person-year outcome ascertainment was observed in this trial (2905 of 3207 possible total person-years), with 75% of bromocriptine-QR subjects and 82% of placebo subjects providing a week 52 plus 30-day follow-up outcome assessment (see Figure 1 for subject disposition and delineation of person-year outcome ascertainment). Reasons given for not completing the study included experiencing an adverse event (605 total [24% bromocriptine-QR, 11% placebo]); withdrawal of consent, identified distinctly as such on the case record termination visit form (259 total [9% bromocriptine-QR, 7% placebo]); and other (419 total [13% bromocriptine-QR, 14% placebo]). A total of 5.5% of patients were lost to follow-up. Six patients died while on the study drug or within 30 days of stopping—4 (0.19%) on bromocriptine-QR and 2 (0.20%) on placebo.

The study population demographics are given in Table 1 and are characteristic of real-world United States T2DM patients. Their mean age was 60 years, 43% were female, and 17% were African American. Hypertension and hypercholesterolemia were prevalent, at 75% and 77%, respectively, and one third of subjects had preexisting macrovascular disease. The majority of subjects (74%) were taking oral antihyperglycemic agents, whereas 14% were receiving insulin with or without an oral agent and 12% were managed only with lifestyle intervention. Metformin was the most commonly prescribed oral antihyperglycemic agent (58%), followed by sulfonylureas (37%). The majority of patients were taking cardioprotective medications (antihypertensives, statins, fibrates, and anticoagulants), with 91% taking ≥1 such agent (Table 2). The average baseline values for HbA1c, fasting glucose, fasting plasma lipid, and blood pressure suggest moderate to good control of these important disease metrics (Table 1). Creatinine level, body mass index, and waist circumference were not unexpected for patients with a duration of diabetes of 8 years (Table 1). Overall, there were no differences in baseline characteristics between the groups that would not be expected by chance (Tables 1 and 2).

**Table 1.** Baseline Demographics and Laboratory Vital Measures of Study Population

Variable	Bromocriptine-QR (N=2054)	Placebo (N=1016)
Mean age	59.5 (±10.2)	60.2 (±9.97)

Duration of diabetes diagnosis, mean (±SD)	7.9 (±7.42)	8.0 (±7.41)

Male sex, n (%)	1141 (56)	598 (59)

White race, n (%)	1381 (67)	698 (69)

Black race, n (%)	348 (17)	168 (16.5)

Hispanic, n (%)	277 (13.5)	131 (13)

Asian, n (%)	22 (1.1)	10 (1.0)

Other, n (%)	26 (1.3)	9 (0.9)

Hypertension, n (%)	1548 (75)	767 (75.5)

Angina pectoris, n (%)	214 (10)	101 (10)

Myocardial infarction, n (%)	186 (9.1)	106 (10.4)

Revascularization surgery, n (%)	204 (10)	128 (13)

Stroke, n (%)	86 (4.2)	63 (6.2)

Hypercholesterolemia, n (%)*	1575 (77)	767 (75.5)

Hypertriglyceridemia, n (%)*	853 (41.5)	422 (41.5)

Current smoker, n (%)	306 (15)	133 (13)

Former smoker, n (%)	802 (39)	419 (41)

*Laboratory and vital measures*		

HbA1c (%), mean (±SD)	7.0 (±1.0)	7.0 (±1.1)

Fasting glucose (mg/dL), mean (±SD)	142 (±41)	141 (±41)

Total cholesterol (mg/dL), mean (±SD)	179 (±43)	177 (±39)

LDL cholesterol (mg/dL), mean (±SD)	98 (±33)	97 (±30)

HDL cholesterol (mg/dL), mean (±SD)	46 (±12)	46 (±12)

Triglycerides (mg/dL), mean (±SD)	181 (±145)	175 (±122)

Systolic blood pressure (mm Hg), mean (±SD)	128 (±14)	129 (±14)

Diastolic blood pressure (mm Hg), mean (±SD)	76 (±9)	76 (±9)

Creatinine (mg/dL), mean (±SD)	1.1 (±0.2)	1.1 (±0.2)

Body mass index (kg/m^2^), mean (±SD)	32.4 (±5.1)	32.3 (±5.1)

Waist circumference (inches), mean (±SD)	41.8 (±5.1)	42.0 (±5.5)

SD, standard deviation; LDL, low-density lipoprotein; HDL, high-density lipoprotein.

* Based on history as assessed by study site investigator.

**Table 2.** Baseline Antihyperglycemic and Cardioprotective Medications of Study Population

Variable	Bromocriptine-QR (N=2054)	Placebo (N=1016)
Diabetes treatment, n (%)		

Diet only	257 (12.5)	114 (11)

One oral hypoglycemic agent	806 (39)	403 (40)

Two oral hypoglycemic agents	686 (33)	323 (32)

Oral agent plus insulin	171 (8)	98 (10)

Insulin only	133 (6)	78 (8)

Not reported	1	0

Antidiabetes medications by agent, n (%)		

Metformin	1209 (59)	581 (57)

Rosiglitazone	233 (11)	111 (11)

Pioglitazone	161 (8)	83 (8)

Sulfonylurea/glinide	759 (37)	392 (39)

Other	29 (1)	19 (2)

Cardioprotective medications by class, n (%)		

ACE inhibitors	994 (48)	477 (47)

Angiotensin II receptor inhibitors	271 (13)	135 (13)

β-Blockers	452 (22)	247 (24)

Diuretics, thiazide	445 (22)	233 (23)

Sulfamides, loop diuretics	166 (8)	89 (9)

Other diuretic*	75 (4)	49 (5)

Calcium channel blockers†	362 (18)	202 (20)

Hmg CoA reductase inhibitor	1165 (57)	594 (58)

Fibrate	157 (8)	78 (8)

Platelet aggregation inhibitors	943 (46)	500 (49)

Cardioprotective medications by number, n (%)		

Taking 1 cardioprotective agent	376 (18)	184 (18)

Taking 2 cardioprotective agents	411 (20)	224 (22)

Taking 3 cardioprotective agents	390 (19)	183 (18)

Taking ≥4 cardioprotective agents	678(33)	345 (34)

* Other diuretics include aldosterone inhibitors, low-ceiling diuretics.

† Calcium channel blockers include dihydropyridine, phenylalkylamine, benzothiazepine.

**Figure 2.**
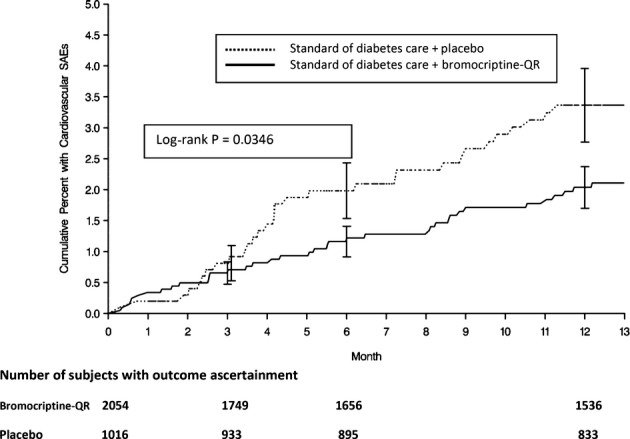
Time to composite end point of cardiovascular serious adverse events including nonfatal myocardial infarction (excluding silent MI), stroke, hospitalization for angina or hospitalization for heart failure, coronary revascularization, and cardiovascular death. SAEs indicates serious adverse events; MI, myocardial infarction.

In this 1-year trial, 72 of 3070 subjects experienced ≥1 of the CV death-inclusive composite cardiovascular end points. Fewer patients experienced this composite end point on bromocriptine-QR (1.9%) versus placebo (3.2%), yielding a statistically significant 39% relative risk reduction (HR 0.61, 95% CI 0.38 to 0.97; *P*=0.02). The between-group difference in cumulative percent of cardiovascular end-point events over the duration of the study was significant in favor of bromocriptine-QR (log-rank test, *P*=0.0346) (Figure 2). Table 3 depicts number of events and hazard ratios for the time to first CV death-inclusive composite cardiovascular end point as well as for each of the components in this composite. Each component of this composite end point had a point estimate <1.0, although none of the hazard ratios for the individual components reached statistical significance.

**Table 3.** Impact of Bromocriptine-QR on CV Death-Inclusive Composite Cardiovascular End Point and Individual Components of the Composite as Well as on the MACE End Point

	Bromocriptine-QR (N=2054), n (%)*	Placebo (N=1016), n (%)*	Hazard Ratio (95% CI)
CV death-inclusive composite cardiovascular end point	39 (1.9)	33 (3.2)	0.61 (0.38 to 0.97)

Myocardial infarction	7 (0.3)	9 (0.9)	0.41 (0.15 to 1.11)

Stroke	5 (0.2)	6 (0.6)	0.44 (0.13 to 1.43)

Hospitalization for angina	9 (0.4)	9 (0.9)	0.52 (0.21 to 1.30)

Hospitalization for heart failure	9 (0.4)	6 (0.6)	0.77 (0.27 to 2.16)

Coronary revascularization	11 (0.5)	8 (0.8)	0.72 (0.29 to 1.80)

CV death	4 (0.2)	2 (0.2)	0.48 (0.07 to 3.43)

Coronary revascularization following a primary end point (ie, CABG after MI)	9 (0.4)	11 (1.1)	0.43 (0.18 to 1.03)

MACE composite—myocardial infarction, stroke, CV death	14 (0.7)	15 (1.5)	0.48 (0.23 to 1.00)

CI, confidence interval; CV, cardiovascular; CABG, coronary artery bypass graft; MACE, major cardiovascular adverse event; MI, myocardial infarction.

* Percentage of events per total number per group: 2054 bromocriptine-QR, 1016 placebo.

In this CV death-inclusive composite cardiovascular end-point analysis (which included revascularization surgery), the time to first composite end point was assessed as the primary end point, and thus coronary revascularization following a primary event such as MI or hospitalization for angina would not be assessed in this Cox analysis. We therefore investigated the influence of bromocriptine-QR treatment on the revascularization surgery incidence rate following a primary CV composite end-point event. Similar to what was seen with coronary revascularization as a primary end point, fewer patients receiving bromocriptine-QR underwent coronary revascularization following a primary CV end point (0.44%) compared with placebo (1.08%), yielding a 57% relative risk reduction in coronary revascularization subsequent to a primary CV event that approached statistical significance (95% CI 0.18 to 1.03) (Table 3).

Men, older patients (>65 years), patients with longer duration of diabetes (>8 years), whites, and patients with preexisting macrovascular disease exhibited a numerically greater number of CV events in the study. However, the CV death-inclusive composite cardiovascular end-point relative risk reduction observed for patients receiving bromocriptine-QR versus placebo was consistent and not influenced by age, race, sex, body mass index, duration of diabetes, or preexisting macrovascular disease (Table 4). That is, treatment by variable interaction was tested, and no significant (*P*<0.05) interactions were observed.

**Table 4.** Impact of Bromocriptine-QR on CV Death-Inclusive Composite Cardiovascular End Point Stratified by Various Demographic Subgroups

	Bromocriptine-QR		Placebo			
				
	N	CV Events, No. People (%)	N	CV Events, No. People (%)	Hazard Ratio (95% CI)	Treatment by Subgroup Interaction, *P* Value
CV-death-inclusive composite cardiovascular end point	2054	39 (1.9)	1016	33 (3.2)	0.61 (0.38 to 0.97)	

Sex						

Male	1141	31 (2.7)	598	26 (4.3)	0.65 (0.39 to 1.09)	0.77
	
Female	913	8 (0.9)	418	7 (1.7)	0.54 (0.19 to 1.49)	

Age, years						

>65	601	21 (3.5)	315	18 (5.7)	0.66 (0.35 to 1.25)	0.88
	
≤65	1453	18 (1.2)	701	15 (2.1)	0.59 (0.30 to 1.17)	

Duration of DM disease, years						

>8	1015	24 (2.4)	518	21 (4.1)	0.62 (0.34 to 1.11)	0.96
	
≤8	1038	15 (1.4)	496	12 (2.4)	0.61 (0.28 to 1.30)	

Race						

White	1381	33 (2.4)	698	26 (3.7)	0.67 (0.40 to 1.13)	0.46
	
Nonwhite	673	6 (0.9)	318	7 (2.2)	0.42 (0.14 to 1.24)	

Preexisting macrovascular disease						

Yes	501	31 (6.2)	271	22 (8.1)	0.80 (0.46 to 1.38)	0.15
	
No	1553	8 (0.5)	745	11 (1.5)	0.34 (0.14 to 0.86)	

Body mass index, kg/m^2^						

≤32	1023	20 (2.0)	522	17 (3.3)	0.63 (0.33 to 1.21)	0.87
	
>32	1031	19 (1.8)	494	16 (3.2)	0.59 (0.30 to 1.15)	

CV indicates cardiovascular; DM, diabetes mellitus.

**Figure 3.**
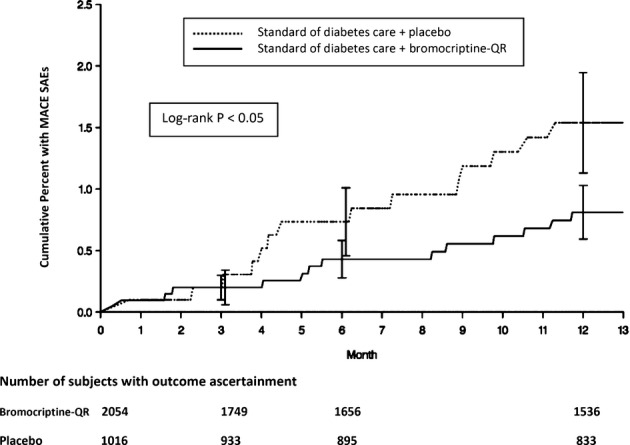
Time to composite end point of nonfatal myocardial infarction (excluding silent MI) or stroke or cardiovascular death (MACE) in ITT population for bromocriptine-QR and placebo groups. SAEs indicates serious adverse events; MI, myocardial infarction; and ITT, intention-to-treat.

An analysis of bromocriptine-QR impact on the MACE (MI, stroke, CV death) end point revealed a 52% relative risk reduction in adverse cardiovascular event rate by bromocriptine-QR exposure (HR 0.48, 95% CI 0.23 to 1.00) (Table 3). The MACE Kaplan–Meier curve (Figure 3) demonstrates a clear separation in MACE cumulative events in favor of those subjects randomized to bromocriptine-QR therapy (log-rank test, *P*<0.05). A sensitivity analysis was conducted on the time to first MACE end point for only the on-treatment population (ie, outcome ascertainment time contributed only while actively on treatment) and similarly revealed a hazard ratio of 0.452 with a 95% CI of 0.205 to 0.996 (*P*<0.05) in favor of bromocriptine-QR.

## Discussion

The current findings of 39% and 52% relative risk reductions in the CV death-inclusive composite cardiovascular and MACE end points, respectively, among bromocriptine-QR versus placebo subjects in the Cycloset Safety Trial corroborate and extend the original observations from this trial on bromocriptine-QR impact on prespecified adverse cardiovascular outcomes that excluded CV death.^5^ Moreover, the effect of bromocriptine-QR on the CV death-inclusive composite cardiovascular end point when stratified by demographic subgroup indicates that the magnitude and direction of the CV disease risk reduction of bromocriptine-QR was similar regardless of age, sex, BMI, race, preexisting CV disease, or duration of diabetes (Table 4). Realizing the limitation of the small number of events in this study, this subgroup interaction analysis was conducted to explore the possibility that a spurious subject demographic among those examined that could influence cardiovascular event rate may have influenced the outcome observed. The results of this analysis suggested no such occurrence. These findings are best appreciated when viewed in the context of the unique study population and trial design employed in the Cycloset Safety Trial, which had few restrictions on enrollment of subjects with comorbid conditions commonly associated with type 2 diabetes (such as CHF or preexisting cardiovascular disease or events). Moreover, approximately one third of the study subjects had preexisting CV disease at baseline; most were taking cardioprotective medications and 2 antihyperglycemic medications (including insulin) prior to randomization.^5^ Consequently, the study population on average was in moderate to good metabolic control, with a median HbA1c of 7.0±1.0%, plasma triglyceride level of 179±137 mg/dL, plasma LDL level of 98±32 mg/dL, and systolic blood pressure of 128±14 mm Hg.^5^ In addition, investigators were instructed to adjust concomitant diabetes treatments (including glycemic-lowering agents) in order to attempt to achieve recommended ADA therapeutic targets. This trial design provided the opportunity to assess the effect of bromocriptine-QR on cardiovascular outcomes in a T2DM population that, on average, was in relatively good metabolic control and against a background of routine diabetes standard of care.

Despite the generally good metabolic control of the population and the instruction for investigators to intervene during the trial to attempt to achieve the glycemic goals of the American Diabetes Association, the placebo arm of the trial had a serious cardiovascular event rate for the CV death inclusive end point of 3.2% and of the MACE end point of 1.5%. These event rates were not different from those of other large T2DM cardiovascular outcomes trials. For instance, in the ADVANCE (Action in Diabetes and Vascular Disease) and ACCORD (Action to Control Cardiovascular Risk in Diabetes) trial standard treatment arms, the MACE rate per 1000 person-years was 21.2 and 19.6, respectively, which is similar to that observed in the placebo arm of the Cycloset Safety Trial (16.4). The Cycloset Safety Trial population was similar to ADVANCE and ACCORD in age, duration of diabetes, and sex, but the average baseline lipids, blood pressure, and HbA1c metabolic parameters were indicative of better control in the Cycloset Safety Trial study population.^8,9^ Intensive glucose-lowering therapies over a 3.5- to 5-year period in the ACCORD and ADVANCE studies did not result in any significant reduction in nonfatal MI, nonfatal stroke, and cardiovascular death (MACE). In contrast, bromocriptine-QR therapy for only 1 year was associated with a 52% relative risk reduction in the MACE end point. These results occurred in the midst of modest improvements in hyperglycemia, hypertension, plasma triglyceride level, and heart rate (modest effects due primarily to the generally good control of these parameters represented in the baseline bromocriptine-QR and placebo populations),^5^ which may have contributed to the CV outcome observed.

The results of analyses of the original prespecified CV composite (including ischemic and nonischemic cardiovascular end points^5^), the present CV-death-inclusive composite, and the MACEs (only ischemic cardiovascular end points) are internally consistent and suggest a positive impact of bromocriptine-QR on adverse CV outcomes in this T2DM population. The results of these analyses and the subgroup interaction analysis conducted in this sudy in total suggest that the observed effect of bromocriptine-QR on CV outcomes is not driven by a particular component of the CV composite studied or by any particular subject demographic investigated. The obvious question that remains is how bromocriptine-QR exposure produced this consistently observed result on cardiovascular outcomes. The Cycloset Safety Trial did not include assessment of any mechanistic data, so any insights on possible mechanisms for this bromocriptine-QR response at the present time must be gleaned from other existing information relating to the impact of bromocriptine on cardiometabolic physiology. The scientific literature on this topic from both preclinical and clinical studies is briefly summarized as follows.

Timed daily bromocriptine administration to insulin-resistant animals normalizes multiple hypothalamic neurophysiological derangements characterized by low hypothalamic dopaminergic tone, elevated ventromedial hypothalamic noradrenergic and serotonergic activity, and elevated paraventricular hypothalamic neuropeptide Y and corticotrophin-releasing factor levels.^10^ This cluster of derangements can potentiate overactivation of the hypothalamic-pituitary-adrenal axis (ie, cortisol release) and of the sympathetic nervous system drive to the liver, adipose, and the cardiovascular system,^10^ as well as increased responsiveness to sympathetic stimulation.^11^ Increased sympathetic activity in adipose results in increased free fatty acid (FFA) mobilization,^12,13^ which in turn can act to induce hepatic very low-density lipoprotein triglyceride synthesis and secretion (postprandially), lipotoxicity, secretion of inflammatory proteins, and insulin resistance.^14^ Increased cortisol and sympathetic drive in the liver increases hepatic glucose output and decreases hepatic glucose disposal, particularly after a meal, which potentiates postprandial hyperglycemia.^15–17^ Increased sympathetic drive to the vasculature induces hypertension^18,19^ and to the heart (including as part of cardiac autonomic neuropathy) can be a major contributor to cardiac disease.^20–26^ Increases in (postprandial) plasma FFA, glucose, and triglycerides as well as in inflammatory proteins and noradrenaline under the vasoconstrictive influence of elevated sympathetic nervous system tone can potentiate generation of vascular reactive oxygen species and increase inflammation, endothelial dysfunction, hypercoagulation, and arteriosclerosis.^27–34^ And increased sympathetic tone itself is a contributor to insulin resistance syndrome.^35–39^

Timed daily administration of bromocriptine to insulin-resistant animals acts centrally to reverse these sequelae of neuroendocrine and metabolic events (overactive hypothalamic pituitary adrenal axis and sympathetic tone and insulin resistance syndrome, respectively) (reviewed in^10^). Finally, and potentially of significant import, bromocriptine therapy of hypertensive, insulin-resistant, arteriosclerotic rats has been shown to reduce arterial stiffness and endothelial nitric oxide synthase uncoupling,^40^ a phenomenon seen with diabetes that results in reduced nitric oxide generation and overproduction of reactive oxygen and nitrogen species in vessel walls that can induce prodigious damage to the vasculature.^41^

It is not known whether the use of bromocriptine to correct these hypothalamic aberrations that precipitate multiple parallel pathophysiological events known to potentiate CV disease is involved in its ability to reduce the MACE end point in the present study. Although not measured in the Cycloset Safety Trial, bromocriptine-QR therapy simultaneously reduced postprandial glucose, FFA, and triglyceride levels in T2DM subjects in other studies.^1,2^ In the Cycloset Safety Trial, bromocriptine-QR produced meaningful reductions in HbA1c and blood pressure among those with elevated HbA1c (≥7.5%) or blood pressure (systolic blood pressure >130 mm Hg) at baseline.^42,43^ And the general sympatholytic effect of bromocriptine in humans has been well appreciated for many years.^44–46^ Although these findings offer intriguing possibilities for identifying pathways by which bromocriptine-QR may produce the results on the cardiovascular end points reported here, much additional research in this area is needed to fully delineate these mechanisms.

The major limitation of this investigation is the inability to fully assess the potential impact of bromocriptine-QR across various demographic subgroups. Because of the number of outcomes, subgroup demographic groupings needed to be broad to assess the impact of bromocriptine-QR on any one of them. Multivariate models of analysis were also not possible on the subgroups investigated. Also, because of the 1-year duration of the study, there were too few deaths to comment on the intervention's influence on total mortality. Nonetheless, the relative risk reduction of the CV death-inclusive composite cardiovascular end point (and the supportive analysis across various subgroups) and of the MACE end point in the bromocriptine-QR-exposed group reaffirms and extends the previous observation of bromocriptine-QR treatment on CV outcomes in T2DM subjects.^5^

## Conclusions

Treatment with bromocriptine-QR reduced the CV death-inclusive composite cardiovascular end point by 39% in T2DM subjects after 1 year of treatment, and this observed relative risk reduction was consistent regardless of age, duration of disease, race, sex, or preexisting CV disease. Furthermore, among such subjects with type 2 diabetes receiving commonly prescribed diabetes therapies, bromocriptine-QR significantly reduced the relative risk for the composite end point of myocardial infarction, stroke, and cardiovascular death (MACE) by 52%.
